# Supplementary material for: A multiple instance learning approach for detecting COVID-19 in peripheral blood smears
Source: PLOS Digit Health. 2022 Aug 19;1(8):e0000078. doi: 10.1371/journal.pdig.0000078 (PMC9931330; doi:10.1371/journal.pdig.0000078)
Supplement: S1 File — (PDF) [file pdig.0000078.s001.pdf]

A multiple instance learning approach for detecting COVID-19 in peripheral blood smears

Colin L. Cooke[1], Kanghyun Kim[2], Shiqi Xu[2], Amey Chaware[1], Xing Yao[2], Xi Yang[2], Jadee Neff[3], Patricia Pittman[3], Chad McCall[3], Carolyn Glass[3], Xiaoyin Sara Jiang[3], and Roarke Horstmeyer*[1,2]

[1]Electrical and Computer Engineering Department, Duke University
[2]Biomedical Engineering Department, Duke University
[3]Pathology Department, Duke University Hospital

June 17, 2022

1 Model Training and Architecture

1.1 Training Infrastructure

To implement the neural network models used throughout this work we used PyTorch [1]. During the training process we applied data augmentation to individual blood cell images across both branches and their respective training regimes. The images were augmented using *Torchvision* [2] with standard horizontal/vertical flipping, random rotations, and color jittering. We tracked performance on the held-out validation set during model training using *Weights & Biases*[3], with the final model used for evaluation being the iteration of the model which performed best on the validation set.

To test our system across all available data, we used repeated stratified k-fold cross validation from *Scikit-Learn* [4] on a patient level based their COVID-19 test result. There were a total of 236 patients, 125 which were labelled positive for COVID-19. We first split our data into a 83%-17% training/testing split, then split the training data again with a 80%-20% split for our final training and validation sets. The proportion of COVID-19 positive patients was approximately balanced within each set, and there was no cross-over of patient data from set-to-set. We rotated through our dataset, for a total of six folds (a single fold consisting of a unique training/validation/test set). Unless otherwise indicated, all performance metrics are reported as the average test-set performance across all six folds, where multiple independent

models were trained from scratch exclusively on the individual folds. This strategy enabled us to test our system on all available data while isolating the test data during the training process.

1.2 Model Details

The single-image model was trained for a total of 800 epochs. To generate validation metrics representative of our task we evaluated our model by taking the average of all outputs across a patient's data (i.e. running every image included within a patient's PBS and taking the average output as the patient prediction). The final model selected was the model which had peak validation performance, which is evaluated at the end of every epoch.

The multi-image branch of our system consists of three steps: feature extraction, attention based aggregation, and classification. For the feature extraction portion of our pipeline we chose the ResNet50 [5] neural network architecture, configured to produce a feature vector, $f_i \in \mathcal{R}^{32}$, for each image i. The attention mechanism used a multilayer perceptron (MLP) and a *tanh* activation to produce a single *importance* value per image followed by a softmax function. The produced feature weights are used to integrate the feature vector. Finally this combined feature vector is transformed into a classification with a single linear layer.

Several components of our multi-image branch were critical to successful performance. The most important component was the use of a unique random image subset generation process for model training, which was not used during inference. Specifically, during model training, we randomly selected a subset of 16 images per patient (from all available per-patient images) to form our model input. This random set of 16 training images changed at each iteration step of model optimization. During model inference, however, we used all available images from each patient (see Fig 2 for the distribution of image count across patients). This random sampling strategy first helped address high memory usage encountered during network training, Second, we hypothesize that it also had the beneficial effect of acting as a regularization strategy (akin to image augmentation), preventing the network from utilizing relatively few images within a patient's PBS image set to drive predictions.

We trained our multi-image model for 2000 epochs. Similar to the single-image model validation performance was evaluated by examining the prediction of the patient's infections status using *all* of their data, and the model weights which achieved peak performance were saved for final evaluation.

Parameter	Single-Image	Multi-Image
CNN Architecture	DenseNet-121	ResNet50
Minimum Learning Rate	1×10^{-5}	3×10^{-5}
Maximum Learning Rate	1	3×10^{-5}
Optimizer	SGD	AdamW [6]
Batch Size	32	8
Images per Sample	1	16

Table 1: Model Training and Configuration Parameters

2 White blood cell classifier

Within the main body of this work we drew relationships between the attention values produced by one of our multiple instance learning (MIL) methods and categories of white blood cells (WBCs). To develop this relationship we first needed to be able to classify each of the individual cells into predefined categories. To enable WBC classification we used a pre-existing open-source dataset produced by Acevedo et al. [7], which used the same model of imaging system (*Cellavision DM9600*) used within our work. Within their dataset they classified WBCs into eight distinct classes: Neutrophils, Eosiophils, Basophils, Lymphocytes, Monocytes, Immature Granulocytes (IG), Erythroblasts, and Platelets. However, we found that within our dataset there were a large number of what are canonically known as *Smudged Cells*, these are WBCs which are broken during the slide making process [8], these cells were not represented within the dataset provided by Acevedo et al. [7]. To compensate for this exclusion we manually labelled 2000 images within our main dataset and included them with the 17,000 images of the other classes.

Using this expanded dataset we trained a convolutional neural network (CNN) to predict the classification of each cell. We followed a similar procedure to the single-image classifier used for detecting COVID-19, using the same set of image augmentations and the ResNet50 [5] network architecture, trained from random initialization. We used the AdamW optimizer [6] with a fixed learning rate of 3×10^{-4}, and trained the model for 100 epochs. We used stratified K-Fold cross validation to ensure equal proportions of each cell type across our training, validation, and test sets of 70%, 15%, and 15% respectively. With this training strategy our model was able to achieve an average classification accuracy across our test set of 92%

3 Scan Data Statistics

To understand the underlying trends within our data we plotted the distribution of WBC types (determined by our automated WBC classifier). Within Fig 1 we can see that the most common cell is the neutrophil, constituting $\sim 35\%$ of the images, while other cell types occur at a

medium to low frequency. We also examined the number of images per-patient, as shown in Fig 2. Here we can see that there is no meaningful difference between image counts between condition (COVID-19 positive vs. negative), however there is a great deal of variation across the entire dataset.

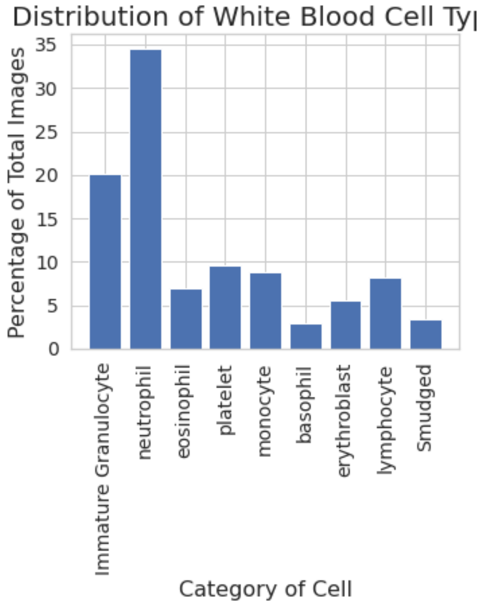

Figure 1: **Distribution of images by white blood cell classification (using automated WBC classifier).**

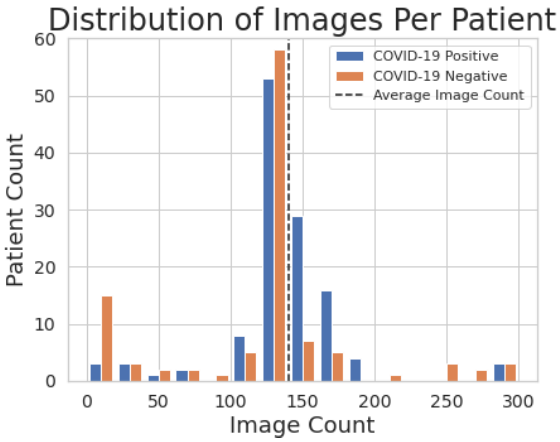

Figure 2: **Number of images per patient across the entire standard dataset.** There was a large degree of variance between patients in image count, with an average of 134 images per patient.

References

[1] A. Paszke, S. Gross, F. Massa, A. Lerer, J. Bradbury, G. Chanan, T. Killeen, Z. Lin, N. Gimelshein, L. Antiga, *et al.*, "Pytorch: An imperative style, high-performance deep learning library," in *Advances in neural information processing systems*, pp. 8026–8037, 2019.

[2] S. Marcel and Y. Rodriguez, "Torchvision the machine-vision package of torch," in *Proceedings of the 18th ACM international conference on Multimedia*, pp. 1485–1488, 2010.

[3] L. Biewald, "Experiment tracking with weights and biases," 2020. Software available from wandb.com.

[4] F. Pedregosa, G. Varoquaux, A. Gramfort, V. Michel, B. Thirion, O. Grisel, M. Blondel, P. Prettenhofer, R. Weiss, V. Dubourg, J. Vanderplas, A. Passos, D. Cournapeau, M. Brucher, M. Perrot, and E. Duchesnay, "Scikit-learn: Machine learning in Python," *Journal of Machine Learning Research*, vol. 12, pp. 2825–2830, 2011.

[5] K. He, X. Zhang, S. Ren, and J. Sun, "Deep residual learning for image recognition," in *Proceedings of the IEEE conference on computer vision and pattern recognition*, pp. 770–778, 2016.

[6] I. Loshchilov and F. Hutter, "Decoupled weight decay regularization," *arXiv preprint arXiv:1711.05101*, 2017.

[7] A. Acevedo, S. Alférez, A. Merino, L. Puigví, and J. Rodellar, "Recognition of peripheral blood cell images using convolutional neural networks," *Computer Methods and Programs in Biomedicine*, vol. 180, p. 105020, 2019.

[8] D. Macdonald, H. Richardson, and A. Raby, "Practice guidelines on the reporting of smudge cells in the white blood cell differential count," *Archives of pathology & laboratory medicine*, vol. 127, no. 1, pp. 105–105, 2003.
