# Supplementary material for: A multiple instance learning approach for detecting COVID-19 in peripheral blood smears
Source: PLOS Digit Health. 2022 Aug 19;1(8):e0000078. doi: 10.1371/journal.pdig.0000078 (PMC9931330; doi:10.1371/journal.pdig.0000078)
Supplement: Attachment — Submitted filename: PlosDigiHealth response.pdf [file pdig.0000078.s002.pdf]

Dear Editor,

Thank you for considering our manuscript, “Deep Optical Analysis of Peripheral Blood Smear Images for Next Generation Screening” (Manuscript ID: PDIG-D-22-00017). We would first and foremost like to express our gratitude to the reviewers for their very constructive and helpful feedback. We are encouraged by their generally positive viewpoints regarding our work, including R1’s thought that “it is well written and describes the methods rigorously often lacking in other ML manuscripts,” as well as R2’s impression that our “study indirectly elucidated the mechanism by which the model detects COVID-19 through perturbation studies. It breaks the conventional perception that the mechanism of ML models are unclear and the universal reliability is low”. The comments and suggested revisions are highly informative, and we have used them to improve the manuscript significantly. Below is a point-by-point response to each of the reviewers’ major concerns, where we also list out the revised manuscript text when appropriate.

Reviewer #1: The manuscript is interesting in its exploration to build a deep learning model to predict Covid from PBS. It is well written and describes the methods rigorously often lacking in other ML manuscripts. The study design is interesting especially the pertubation studies which investigates the cell type of relevance.

Having said this, there are certain shortcomings of this study which need to be addressed to answer some key questions and to make such a model utilizable in the clinical setting.

1. It is strongly suggested to the authors to reword the manuscript title. It currently makes no mention of what exactly was investigated in this study let alone give any indication that the use case was Covid. Secondly, the phrase "next generation screening" is also confusing as no mention of using the model as a screening test is made in the manuscript. NGS also has strong word association with the genomics domain and can be confusing for readers and clinicians who might have different expectations going in when they read this manuscript.

- We agree with the reviewers' concerns, to address this potential confusion we have modified the title in line with the reviewers' suggestion. The title is now: “A multiple instance learning approach for detecting COVID-19 in peripheral blood smears”

2. Study cohort selection- Section 2.1 describes the standard and challenge cohorts and the patient populations from whom these smears were used. It is unclear why the rest of the 47% standard cohort was included if only 53% pts were RT-PCR positive. Moreover what is the difference then between the 47% standard cohort and the challenge cohort who too tested RT-PCR negative.

- Thank you for raising this question and we apologize for our unclear presentation. To clarify the reasons why two cohorts were collected, the section in question has been modified. We now state that the challenge set was specifically collected to test if the system can distinguish between COVID-19 and other respiratory illnesses, while the

collection of the larger COVID-19 negative portion of the standard cohort was necessary to enable the development of the machine learning system.

3. It is strongly recommended to include the data preprocessing section from supplemental to the main manuscript before Section 3.2. Understanding this is key in addition to the model parameters and architecture.

- We thank the reviewer for this great suggestion. The data preprocessing paragraph has been placed between *data collection* and *machine learning system* subsections, to make the methods section more readable as follows.
- We applied the same kind of data preprocessing and augmentations to all images used within this study. To prepare the data for processing by our neural networks we cropped and normalized the images. The cropping was done by taking the inner 240 × 240px from the original 360 × 360px image. We normalized the images by calculating a dataset wise mean and standard deviation for each color channel (red, blue, and green), then subtracting the mean and dividing by the standard deviation. To augment our dataset we applied, in order, random horizontal and vertical flipping, random rotation, and color jittering. These augmentations were implemented using TorchVision with parameters reported in Table 1. The aforementioned cropping step was done after the augmentations had been applied. During our perturbation experiments, we added a final masking step to our augmentation pipeline, replacing content within the mask area with zeros.

Table 1. Data augmentation and pre-processing parameters (see Torchvision [31] for implementation details).

Parameter	Value
Random Horizontal Flipping	50% probability
Random Vertical Flipping	50% probability
Color Jitter	$\pm[0,5]\%$ Brightness<br>$\pm[0,5]\%$ Contrast<br>$\pm[0,5]\%$ Saturation<br>$\pm[0,5]\%$ Hue
Random Rotation	Uniformly sampled from $[-45, 45]$
Center Crop	Center 240×240px

Transformations are applied in the order they appear within the table. The same transformations were applied in both the single and multi-image models.

4. Was this model validated on an external PBS dataset acquired outside of the primary medical center mentioned in this study? Is it a possibility to perform it? If not, please mention this as a limitation in discussion. Evidence for generalizability is commonly expected from ML studies.

- We agree with the reviewer that our method of data collection will potentially limit the ability of our system to scale beyond data collected in the single environment we used

for data collection. We have adjusted our discussion section to make it clear that such a limitation may exist, and that it would be beneficial to have a cross-institutional data collection strategy moving forward, which will directly enhance and test the generalization capabilities of our system moving forward.

5. The biggest critique of this study is its inability to explain what cell features does the ensemble model recognize and weigh heavily in its inference. Correlation at the cell type level is very generic and do not indicate why it would be unique to a Covid infection. E.g. Neutrophils which are most highly associated with the model, increase commonly across a variety of bacterial and viral infections. The authors are strongly recommended to provide a posthoc explanation of what features the model relies on to make its decision e.g. inclusion bodies, changes nucleocytoplasmic ratio etc

- We thank the reviewer for their very insightful suggestion here. We all agreed whole-heartedly that a posthoc analysis would be a very useful exercise. To attempt the first step at such a post-hoc explanation, we had a trained pathologist perform additional visual analysis of the cells determined “most important” by our machine learning system. In this analysis, a trained pathologist examined the 100 cells, looking for the presence of specific abnormalities (Dohle Bodies, Vacuoles, Hyposegmentation (either band formation or two discrete segments), Hypercondensation of Chromatin, Nuclear Bridges, Toxic Granulations, Nuclear Blebs, Abnormal Nucleus to Cytoplasm Ratio, Hypersegmentation, Inclusion Bodies, and Green Crystals). After the analysis was complete we aggregated the data and looked at the occurrence rate of specific abnormalities across our COVID-19 positive and negative groups. We found that specific kinds of abnormalities, traditionally associated with infection, occurred significantly more often in the cells our system indicated as being “important” and that were from our COVID-19 positive group. We therefore can infer that our machine learning system may in part be using these abnormalities as indicators for a COVID-19 infection.
- The text of Section 2.3 has been updated to reflect these findings as follows.
- To better understand potential cellular features which may be influencing how the system gauges the importance of individual cells we performed a visual analysis of a subset of the cells (100 in total) deemed ”most important” by the system, for both the COVID-19 negative and positive cohorts. We looked for the presence of a discrete set of well known abnormalities affecting blood cells, these were: Dohle Bodies, Cytoplasmic Vacuoles, Hyposegmentation (either band formation or two discrete segments), Hypercondensation of Chromatin, Nuclear Bridges, Toxic Granulations, Nuclear Blebs, Abnormal Nucleus to Cytoplasm Ratio, Hypersegmentation, Inclusion Bodies, and Green Crystals. Each individual image of a cell was examined by a trained pathologist to determine which, if any, of the above abnormalities were present. We then compared the occurrence rate of abnormalities, by type, across the COVID-19 positive and negative cells. The results are shown in Fig 5.

  Using logistic regression, we predicted the presence of each of these attributes within each cell from the COVID test status (negative vs. positive). We found that three of target attributes were present at a significantly higher rate for cells from COVID positive

patients, they were Dohle Bodies (β = 2.85, 95%CI[1.17, 5.77], z = 2.68, p = 0.007), Cytoplasmic Vacuoles (β = 2.38, 95%CI[0.65, 5.31], z = 2.21, p = 0.027), Hyposegmentation (β = 1.29, 95%CI[0.46, 2.16], z = 2.99, p = 0.003). All three of these abnormalities are indicative of infection. Therefore we can infer that our machine learning system may at least in part use these kinds of abnormalities as indicators for a COVID-19 infection. There was either no significant difference (p >= 0.05) or too few observations to make a statistical conclusion with our 100 cell sample for the remaining abnormalities.

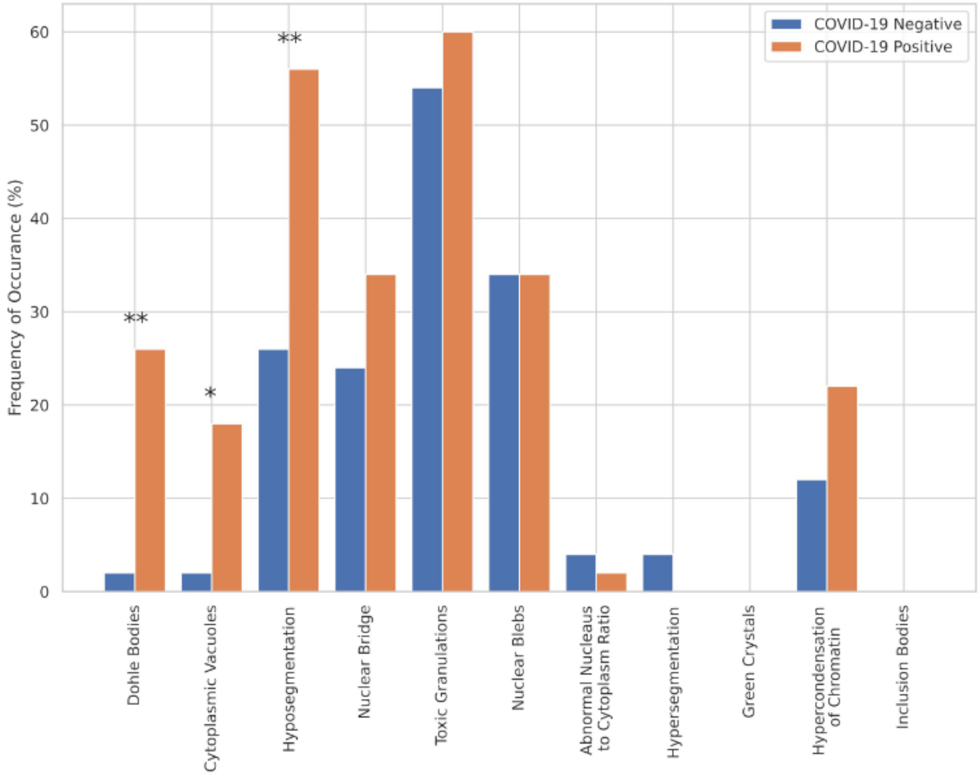

Fig 5. The occurrence rate of abnormalities between COVID-19 negative and positive cells. Significance is indicated on the three kinds of abnormalities that were found to significantly differ in frequency between condition.

6. An understanding of how long such cell specific Covid associations and morphological changes can be identified is desirable. For instance, what happens when a patient recovers from Covid and has a followup blood exam a month after. Will this model still infer the PBS to be Covid positive if the cell changes haven't returned to baseline? It is suggested to examine model performance on longitudinal data if possible. This would really add strong clinically actionable insight to the current study.

- We share the reviewer's interest in the longer-term effects of COVID-19 on blood, and the potential to use a model such as the one outlined in the paper, to begin to understand those effects. Unfortunately due to the data collection strategy used within this work we are limited to a "snapshot view" of each patient through their peripheral

blood smear. Such a longitudinal analysis would be very interesting to study in future work and agree that it could provide clinical value.

- To improve the readers' understanding of the limitations of our work and outline this new future direction we have added additional paragraphs to section 4 which review these concepts as follows.
- In this paper, we present a MIL-based method to accurately diagnose the COVID-19 disease at a per-patient level from high-resolution morphological information across many blood cells and cell types. Besides the final aggregated decision, the proposed attention mechanism also provides cell-type importance, which can help pathologists to build valuable insights on which cell types are more diagnostically relevant. Moreover, by evaluating how different perturbations to our image dataset can affect the diagnosis results, we also studied which morphological features are more critical to the screening, opening a window into improving the explainability of machine learning approaches.

  While our study shows promising outcomes, there are a few limitations of this pilot study. For example, the data collected from a single site for a short period of time. We hope that our new data-driven aggregation strategy can be applied for larger data collected from more diverse regions around the world, to serve as a starting point for future algorithmic development to understand the hematological impact of COVID-19 and other blood-related diseases.

  The main limitation of this study is that the data collected represents a "snapshot" view of the patient's status. The peripheral blood smears were collected once, with no follow-up of additional measures afterwards. That means that we are unable to expand on our system's capabilities to begin to understand if we can determine the recovery trajectory of the patient, or predict the severity of the disease. Furthermore, we are limited to a peripheral blood smear per patient, so we cannot use our system to understand how the appearance of blood cells may change over time in response to an infection. In addition to limitations stemming from the method in which the samples were collected, there are potential limitations arising from the location of collection. In similar past work using data from a single institution, or medical device, may cause inherent bias in the data, making the method unable to perform effectively outside the environment it was developed in. With the data currently available, we are unable to test the generalization capabilities of our system and therefore cannot gauge if it would be effective in alternate environments.

  These limitations all lead towards potentially promising future work, where a more longitudinal and cross-institutional study of blood can be done, both by collecting more than one sample over time, and by collecting other measures in addition to peripheral blood smears (such as time to recovery, or measures of disease severity) to further understand the limits of our system's capabilities.

Reviewer #2: This study suggest a multi-instance learning-based approach to aggregate high-resolution morphological information across many blood cells and cell types. This can be used as a part of clinical decision support system.

Overall descriptions and figures about the workflow of the proposed system are well expressed. In particular, it explains the meaning of the results in detail. This enhances the persuasion of the proposed system. By analyzing the importance of each cell type, cells with higher and lower infection were identified. Also, this study indirectly elucidated the mechanism by which the model detects COVID-19 through perturbation studies. It breaks the conventional perception that the mechanism of ML models are unclear and the universal reliability is low.

Minor comment:

1. The abbreviation of SIL is not indicated. Also, in the figure 2, if there is no problem changing the 'branch' term to 'learning', I recommend that you change it.

- We apologize that we did not indicate the abbreviation of SIL properly. We updated the text with Single Instance Learning (SIL). We thank the reviewer for their suggestions, the figures have been modified to include the more appropriate term “learning” instead of “branch”.

2. A sentence often extends over 3 to 4 lines, making it difficult to grasp the context. In many cases, the results and reasons are expressed in one sentence, and I recommend you to divide the sentences.

- We are sorry for the confusion coming from the long sentences. We edited these longer sentences throughout the manuscript to improve their grammatical clarity.

3. Method section: Please describe in more detail how the attention mechanism was applied to the model.

- We appreciate about reviewer’s suggestion. We added more details in the Method section as follows.
- The attention mechanism used a small multilayer perceptron (MLP) with one hidden layer of size 64 and a tanh activation to produce a single importance value per image (a_i). Each importance value is placed into a softmax function to produce a set of feature weights W, such that w_i = e^(a_i)/ $\sum_{j=1}^{N}$ e^(a_j) and $\sum_{j=1}^{N}$ w_j = 1. These weights are used in conjunction with the extracted features to create a final per-image set feature vector.

Section 2.2: The description in Figure 2 refers to the attention mechanism, but it is difficult to understand in Figure itself. Please modify the figure more intuitively. Also, there is not enough description of the formulas in the figure (ex. Ma, Mc...).

- We agree with the reviewer's assessment and have made adjustments to the figure and surrounding text to better communicate the method. While the figure itself is not significantly changed we have added a substantial amount of text reviewing the contents of the figure and outlining the notation/formulae used within it. We hope these adjustments clarify what the figure is attempting to convey and thank the reviewer for their observations around the lack of previous clarity.
- The figure encapsulates the various discrete steps involved for each branch under distinct operators. Within the Single-Image Learning branch the main operator is the Single Image Model (M_s), which takes in a single patient image (I_i), and the model parameters (θ_s) to produce single-image predictions. The median of these predictions is then fed into the ensembling step. For the Multi-Image Learning branch we first apply a feature extraction model (M_f) to each patient image (I_i) using parameters θ_f. Each of these feature vectors (f_i) are used by the attention mechanism (M_a) alongside the learned parameters for the attention mechanism, θ_a, to produce a feature weight vector (W). Next the feature vectors are weighted by elements of the feature weight vector and a weighted sum of the features is found (F). Finally the patient prediction is made by applying a final learned model (M_c) to the weighted feature vector (F) with parameters θ_c to create the patient prediction for this branch.
